# Lack of Structural Variation but Extensive Length Polymorphisms and Heteroplasmic Length Variations in the Mitochondrial DNA Control Region of Highly Inbred Crested Ibis, *Nipponia nippon*

**DOI:** 10.1371/journal.pone.0066324

**Published:** 2013-06-21

**Authors:** Xue-Lian He, Chang-Qing Ding, Jian-Lin Han

**Affiliations:** 1 College of Nature Conservation, Beijing Forestry University, Beijing, China; 2 CAAS-ILRI Joint Laboratory on Livestock and Forage Genetic Resources, Institute of Animal Science, Chinese Academy of Agricultural Sciences (CAAS), Beijing, China; 3 International Livestock Research Institute (ILRI), Nairobi, Kenya; Vanderbilt University Medical Center, United States of America

## Abstract

The animal mitochondrial DNA (mtDNA) length polymorphism and heteroplasmy are accepted to be universal. Here we report the lack of structural variation but the presence of length polymorphism as well as heteroplasmy in mtDNA control region of an endangered avian species – the Crested Ibis (*Nipponia nippon*). The complete control region was directly sequenced while the distribution pattern and inheritance of the length variations were examined using both direct sequencing and genotyping of the PCR fragments from captive birds with pedigrees, wild birds and a historical specimen. Our results demonstrated that there was no structural variation in the control region, however, different numbers of short tandem repeats with an identical motif of CA_3_CA_2_CA_3_ at the 3′-end of the control region determined the length polymorphisms among and heteroplasmy within individual birds. There were one to three predominant fragments in every bird; nevertheless multiple minor fragments coexist in all birds. These extremely high polymorphisms were suggested to have derived from the ‘replication slippage’ of a perfect microsatellite evolution following the step-wise mutational model. The patterns of heteroplasmy were found to be shifted between generations and among siblings but rather stable between blood and feather samples. This study provides the first evidence of a very extensive mtDNA length polymorphism and heteroplasmy in the highly inbred Crested Ibis which carries an mtDNA genome lack of structural genetic diversity. The analysis of pedigreed samples also sheds light on the transmission of mtDNA length heteroplasmy in birds following the genetic bottleneck theory. Further research focusing on the generation and transmission of particular mtDNA heteroplasmy patterns in single germ line of Crested Ibis is encouraged by this study.

## Introduction

**Figure 1:**
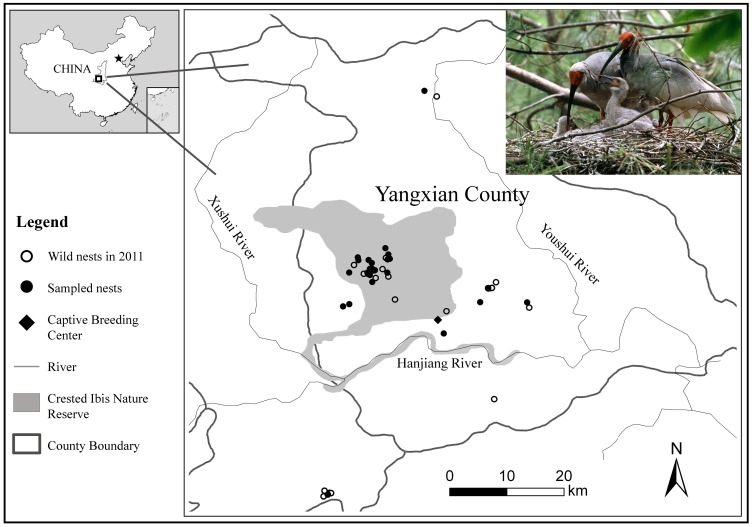
The current distribution range of the wild population, locations of the 43 nests banded in 2011 and 25 nests selected for this study in the wild as well as the Captive Breeding Center of Crested Ibis. Photo courtesy of Jiao Jingquan.

The Crested Ibis (*Nipponia nippon*) was widely distributed in Northeast Asia, however, the overexploitation and destruction of habitats led to the extinction of its Korean, Japanese and Russian populations in the wild in the late 20^th^ century [1], [2], [3]. In May 1981, a remnant population consisting of two pairs of adults and three nestlings was re-discovered in Shaanxi Province, China [4]. Since then, intensive conservation efforts have been made to restore this species in the wild with up to 600 birds by 2007 [5] and to conserve it in captivity with 600 birds by 2009 [6] (Figure 1; also see Fig. 2 in [5] as well as Fig. 1 and 3 in [6] for more details). This species was once recognized as “Critically Endangered” but has been considered as “Endangered” since 2000 in the IUCN Red List of Threatened Species [7]. Understanding and identification of survived genetic variations are therefore critical for the long-term protection and management of all existing populations recovered from such limited founders which must have made this species to be highly inbred.

**Figure 2:**
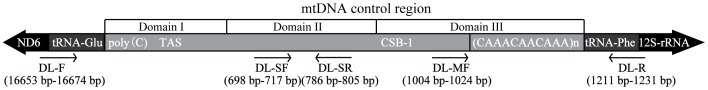
Structure of mtDNA control region and position of primers designed for this study. TAS = termination associated sequences, CSB = conserved sequence block. The primer positions are scored based on the complete mtDNA sequence of Crested Ibis deposited in GenBank (Accession no. NC_008132).

**Figure 3:**
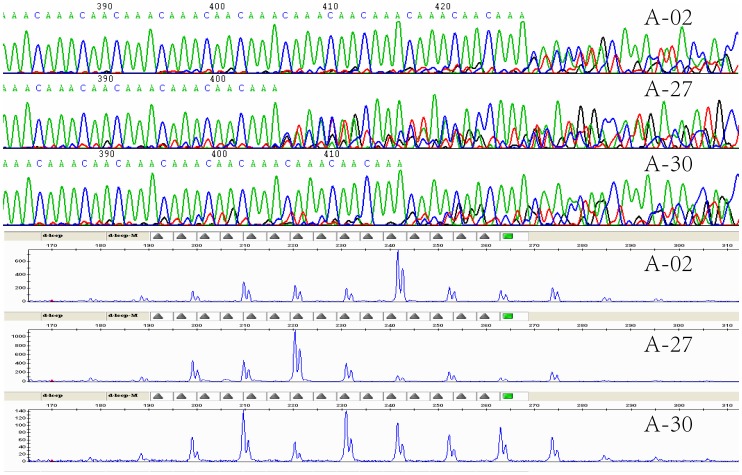
Partial sequencing chromatographs and peak distributions of genotyping of representative samples showing length polymorphism and heteroplasmy. A-27 and A-02 had predominant fragments at 221 bp and 243 bp while their sequencing profiles showed relatively clear chromatograms carrying nine and 11 repeats, respectively. Sample A-30 had at least two predominant peaks at 210 bp and 232 bp whilst its sequencing chromatogram included overlapped signals at an interval of two repeats.

The polymorphic length heteroplasmy (coexistence of two or more types of mtDNA genomes in different lengths within one individual) in mtDNA was reported in a wide range of animals, such as fruit flies [8], [9], crickets [10], [11], [12], lizards [13], fishes [14], [15] and elephant seals [16]. In avian species, mtDNA length heteroplasmy was detected in rails (*Rallus elegans* and *R. longirost*) [17] and mourning dove (*Zenaida macrour*) [18] by restriction analysis of purified mtDNAs. Sequencing of PCR products revealed the length polymorphism and heteroplasmy in mtDNA control region of 15 species of the order Charadriiformes [19] and two vulture species *Gypaetus barbatus* and *Neophron percnopterus* [20]. Intensive studies on the transmission of mtDNA genomes suggested a theory of ‘genetic narrow bottleneck’ following the proportion shift of mtDNA structural heteroplasmy between mother and offspring as well as between offspring in cattle, mice, human and salmon [21], [22], [23], [24], [25], [26], [27], [28], [29], [30], [31], [32]. To our knowledge, the transmission of either length or structural heteroplasmy in mtDNA of birds remains to be examined.

Previous studies revealed an extremely low genetic diversity in the current populations of Crested Ibis both in the wild and captivity using mtDNA control region sequences and microsatellite DNA markers [33], [34]. Sequences of domains II and III at 3′-end of the control region generated using PCR and cloning protocols were reported to have an intra-individual length heteroplasmy in the domain III. The heteroplasmy was defined by two different DNA structures of either a simple tandem repeat of (CA_3_CA_2_CA_3_)n or a complicated repeat (CA_3_CA_2_CA_3_)_4_CA_3_C**G**ACA_3_(CA_3_CA_2_CA_3_)_2_C**G**A_2_CA_2_CA_3_(CA_3_CA_2_CA_3_)_3_ [34]. However, the distribution pattern and inheritance of the heteroplasmy were not fully investigated. In this study, both direct sequencing of PCR products and genotyping of PCR fragments were applied to investigate structural variation and length heteroplasmy in the mtDNA control region of the highly inbred Crested Ibis.

## Materials and Methods

### Sampling and DNA Extraction

Blood samples from 28 birds and both blood and feather samples from five individuals were collected in 2010 from Shaanxi Crested Ibis Breeding Center, Yangxian County, Shaanxi Province, China. These 33 captive adult Crested Ibis belonged to three families following pedigree records (Table 1). There were 23 individuals across three generations in family No. 1 and five individuals each across two generations in families No. 2 and No. 3. Another 27 feather samples were collected from nestlings with one each of 24 nests and three of one nest in the wild during banding season in 2011 in Shaanxi Hanzhong Crested Ibis National Nature Reserve (Figure 1). One foot pad sample was derived from a specimen, which was acquired from Xi’an of Shaanxi province in 1915 and is preserved in the State Zoological Museum of China in Beijing. This study was fully endorsed by both the Center and the Reserve and finally approved by the Institutional Animal Care and Use Committee (IACUC) of the College of Biological Sciences and Technology, Beijing Forestry University. All the sampling activities were conducted in collaboration with technical staff of the Center and the Reserve. Maximal care was taken during either bleeding for adults or banding for nestlings as part of the routine practices which have been applied to monitor the captive and wild flocks. The foot pad sample was donated by the Museum. These samples were strictly stored and used for research purpose only following specific collaborative agreements between the institutions.

**Table 1:** Number of repeats in predominant fragments in 33 captive Crested Ibis.

Family	Mating group	Parent		Offspring	
		Female	Male		
No. 1	1	A-05∶11	A-04∶11	Female	A-06∶12 A-08∶11 A-10∶9 A-20∶12 A-33∶9
				Male	A-18∶12 A-28∶11
	2	A-08∶11	A-07∶11	Female	A-12∶11
				Male	A-24∶11
	3	A-10∶9	A-09∶13	Male	A-21∶11 A-23∶9
	4	A-17∶10	A-18∶12	Male	A-16∶11, 12, 13
	5	A-33∶9	A-30∶8, 10	Female	A-11∶9
				Male	A-27∶9 A-29∶9 A-31∶9 A-32∶8
No. 2	6	A-26∶9	A-25∶10	Female	A-19∶9
				Male	A-13∶8 A-15∶8
No. 3	7	A-01∶11	A-03∶11	Female	A-14∶11
				Male	A-02∶11 A-22∶11

ID of individual is numbered as A-01 to A-33.

The blood samples were collected from wing vein and mixed with same volume of anticoagulant buffer (0.1 M Tris-Cl, 0.1 M EDTA and 1% SDS, pH 8.0). Feather samples were kept in absolute alcohol. All the blood and feather samples were stored at −80°C. The foot pad sample was kept in a sealed envelope at room temperature until DNA extraction. After removal of the alcohol, the calamus (approximately 2 cm) of preserved feathers was washed twice with 1×sodium chloride-Tris-EDTA buffer (pH 8.0) and cut into pieces to get the pulp tissue inside the calamus. Total genomic DNA was extracted from all the blood, pulp tissue and foot pad samples using a conventional phenol/chloroform protocol [35]. After being qualified and quantified using a NenoDrop-1000 spectrophotometer (NanoDrop Technologies Inc., Wilmington, USA), the DNAs were adjusted to around 100 ng/µl and stored at −20°C.

### Verification of Pedigree Records of the Three Families Using Microsatellite DNA Markers

Eight microsatellite DNA markers (NN01, NN03, NN04, NN12, NN16, NN21, NN25 [33] and NnNF5 [36]) were selected to genotype the 33 pedigreed Crested Ibis for verification of their genetic relationship. All forward primers were labeled by fluorescent dyes and similar PCR conditions were followed for genotyping using an ABI 3130×l Genetic Analyzer (Applied Biosystems, Foster City, CA, USA) with detailed procedures described in later section.

### Amplification of the Control Region and Direct Sequencing

Ten captive and 27 wild birds were chosen to sequence the complete control region. The 10 captive individuals included six parental birds of the first generation of the three families and four additional partners in the second generation of the family No. 1 (Table 1). The complete mtDNA sequence of Crested Ibis (GenBank accession number NC_008132 at http://www.ncbi.nlm.nih.gov/nuccore/NC_008132) was referred to design primers DL-F (5′-GTA AGT CAT AGC CAT TCC TGC T-3′) and DL-R (5′-TTG TCC TTG GGT GCG AGA ATG-3′) (Figure 2) for the amplification of a fragment around 1290 base pairs (bp) covering the complete control region.

The amplification was performed in a 50 µl volume containing around 100 ng DNA, 3.0 units of *EasyTaq* DNA polymerase (TransGen Biotech, China), 5 µl of 10×*EasyTaq* Buffer, 4 µl of dNTPs (2.5 mM, Tiangen Biotech Co. Ltd., China) and 1 µl each of the forward and reverse primers (10 pmol/µl). Thermal cycling was performed using a GeneAmp PCR system 9700 thermal cycler (Applied Biosystems) started with 5 min at 94°C followed by 30 cycles of 40 s at 94°C, 30 s at 64°C and 90 s at 72°C, and finished by a final extension at 72°C for 7 min. Three micro-liters each of the PCR products were run in a 1.5% agarose gel and visualized by GoldView™ Nucleic Acid Stain (SBS Genetech Co. Ltd., China) to confirm their quality and quantity. A total of 33 PCR products of captive and wild birds were purified and directly sequenced using the DL-F primer and two additional sequencing primers of DL-SF (5′-CGT TGG TCC TCA GGA ATT AA-3′) and DL-SR (5′-TAG GGT TAG TGG ATA CAC CA-3′) (Figure 2) for the complete control region including a repeat fragment. The remaining four captive samples were sequenced using the two PCR and two sequencing primers for complete amplicons.

PCR products amplified using the DL-F and DL-R primers from the remaining 23 captive individuals were only sequenced using the DL-SF primer for their last partial control region covering the repeat fragment. The single foot pad sample was amplified using the DL-SF and DL-R primers and directly sequenced using the DL-SF primer for the repeat fragment as well.

The BigDye® Terminator v3.1 Cycle Sequencing Kit (Applied Biosystems) was applied for sequencing reactions that were visualized on an ABI3730 Sequence Analyzer by the Beijing Sunbiotech Co. Ltd. (China). The raw data was manually edited using Chromas version 2.23 (http://www.technelysium.om.au/chromas,html) and aligned along with all sequences retrieved from the GenBank or relevant publications for comparison with MEGA5 software [37].

### Genotyping of the Length Polymorphism and Heteroplasmy in Control Region

To detect the size variations in the repeat fragment, an additional forward primer DL-MF (5′-TCA CAT ACG CGC GCA CAA ACA-3′) (Figure 2) was designed according to the newly generated sequences from this study and labeled by a 6-FAM florescent dye at its 5′-end. It was applied together with the DL-R primer for the amplification of around 250 bp repeat fragment in the domain III among all 61 samples. The amplification was replicated in each DNA sample and performed in a 50 µl volume as above except 2.0 units of *Taq* DNA polymerase (Tiangen Biotech Co. Ltd., China) were used. Thermal cycling was started with 5 min at 94°C followed by 30 cycles of 40 s at 94°C, 30 s at 65°C and 30 s at 72°C, and completed by a final extension of 7 min at 72°C. Two micro-liters of the PCR product were mixed with 7.75 µl deionized formamide and 0.25 µl GeneScan™ 500 LIZ™ Size Standard (Applied Biosystems). This mixture was loaded on the ABI3130×l Genetic Analyzer and fluorescence signal data was collected using Data Collection v3.0 and then analyzed using GeneMapper v3.7 (Applied Biosystems).

## Results

### MtDNA Sequence Analysis

Four sequences of captive birds which were amplified using the DL-F and DL-R primers and sequenced using the DL-F, DL-R, DL-SF and DL-SR primers covered a repeat fragment ranging from the 1072^nd^ nucleotide (scored according to JX853815) in the control region to the beginning of tRNA-Phe region. They all had 1179 nucleotides including 1071 bp remaining control region, 58 bp long segment covering the complete tRNA-Glu region and 50 bp partial tRNA-Phe region. There was no polymorphism present within this 1179 bp long amplicon across these four samples, therefore a single representative sequence was deposited to the GenBank (accession no. JX853815). The control region of 1071 bp from the remaining 33 captive and wild samples shared this representative sequence.

Compared to the reference sequence of complete mtDNA of Crested Ibis (NC_008132), all 37 control region sequences in 1071 bp carried a 19 bp long motif of 10Cs+T+8Cs starting from their 23^rd^ nucleotide while the reference sequence carried only 9Cs, which were probably too difficult to be fully sequenced through. Our new data therefore filled this gap in the reference sequence and confirmed the presence of the interrupted poly(C) structure located at the 5′-end of mtDNA control region of Crested Ibis. A similar structure was identified across several bird groups such as Accipitriformes [20], [38] (AF380305 for *Buteo buteo*; AY542900 for *Gypaetus barbatus* and AY542899 for *Neophron percnopterus*) according to the International Ornithology Committee World Bird List v 3.3 [39], Galliformes [40] (GU187969 for *Tragopan caboti*) and Sphenisciformes [41] (AF272143 for *Pygoscelis adeliae*).

### Length Polymorphism and Heteroplasmy

The repeat fragment was detected in the four sequences amplified by the DL-F and DL-R primers and sequenced by the DL-F, DL-R, DL-SF and DL-SR primers. They all carried an identical repeat motif with 11 nucleotides in (CA_3_CA_2_CA_3_)n at the 3′-end of mtDNA control region. The remaining 33 captive and wild samples which were sequenced using the DL-F, DL-SF and DL-SR primers and the additional 23 captive and wild samples as well as the single historical foot pad sample which were sequenced only with the DL-SF primer shared the same repeat motif. This motif was also present in nine copies in the complete mtDNA sequence of Crested Ibis (NC_008132) and it was identical to the major motif reported by Zhang et al. [34]. Further examination of all 61 sequencing chromatograms generated using the DL-SF primer revealed different numbers of the repeat motifs among individuals (length polymorphisms) and also within individuals (length heteroplasmy) (Figure 3). Additional sequencing profiles of the DL-R primers confirmed our observation. Similar pattern was observed by Zhang et al. [34] and 6, 10 and 11 copies of the motif were identified among and within samples [42]. Clear heterogeneous sequencing profiles, resulted from intra-individual length heteroplasmy, were mostly detected after nine repeated motifs. Therefore it was not possible to determine the exact sizes of the repeat region that caused the variable length of the control region and/or mtDNA genome.

MtDNA length heteroplasmy due to long repeated sequences ranging from around 78 bp to 2 kb was first identified and genotyped using restriction analyses [8], [9], [43], [44], [45], [46]. Following direct sequencing of PCR products of mtDNA control region, Ludwig et al. [14] confirmed the presence of such length heteroplasmy in 12 out of the 19 sturgeon species determined by one to seven central repeat units of 74 to 83 bp long fragments. After discovering a 75-bp tandem repeat segment, Munwes et al. [47] evaluated the length heteroplasmy in the eastern spadefoot toad (*Pelobates syriacus*) based on the number of distinct bands in PCR amplified repeat region on the agarose gel. Nesbø et al. [15] applied procedures of both cloning and direct sequencing of PCR products to assess the length variation and heteroplasmy of a tandem array of 10 bp repeats at the 5′-end of D-loop in two percid fish species. It is clear that the accuracy of these methods mostly depends on the degree and complexity of the heteroplasmy, especially for the low level heteroplasmy of short repeat motifs. Cloning and direct sequencing as well as fragment size analysis were used in interpreting the length heteroplasmy defined by several short repeat sequences in human mtDNA control region [48], [49], [50].

To validate the length polymorphisms and heteroplasmic length variations observed among the directly sequenced PCR products as described above, the size variations and their distribution patterns of the repeat fragment were further genotyped. It was evident that all genotyping profiles carried multiple peaks at least ranging from six to 14 repeats at different heights among and within all birds in this study. Comparison of sequencing and genotyping profiles across every sample confirmed the length polymorphisms among the samples and length heteroplasmy within samples, both were attributed to highly variable numbers of the same repeat motif in (CA_3_CA_2_CA_3_)n. However, one to three predominant peaks at similar heights (fluorescent intensity) in genotyping profiles and relatively clear sequencing chromatograms carrying one to three motifs with respective number of repeats were observed within all samples (see Figure 3). On the other hand, all the samples had relatively low sequencing signals and minor peaks at a regular internal of the 11 bp identical repeat. The same patterns were detected in both sequencing and genotyping results of duplicated blood and feather samples of five birds, demonstrating that the length polymorphisms and heteroplasmy may not be tissue specific in Crested Ibis mtDNA control region.

**Figure 4:**
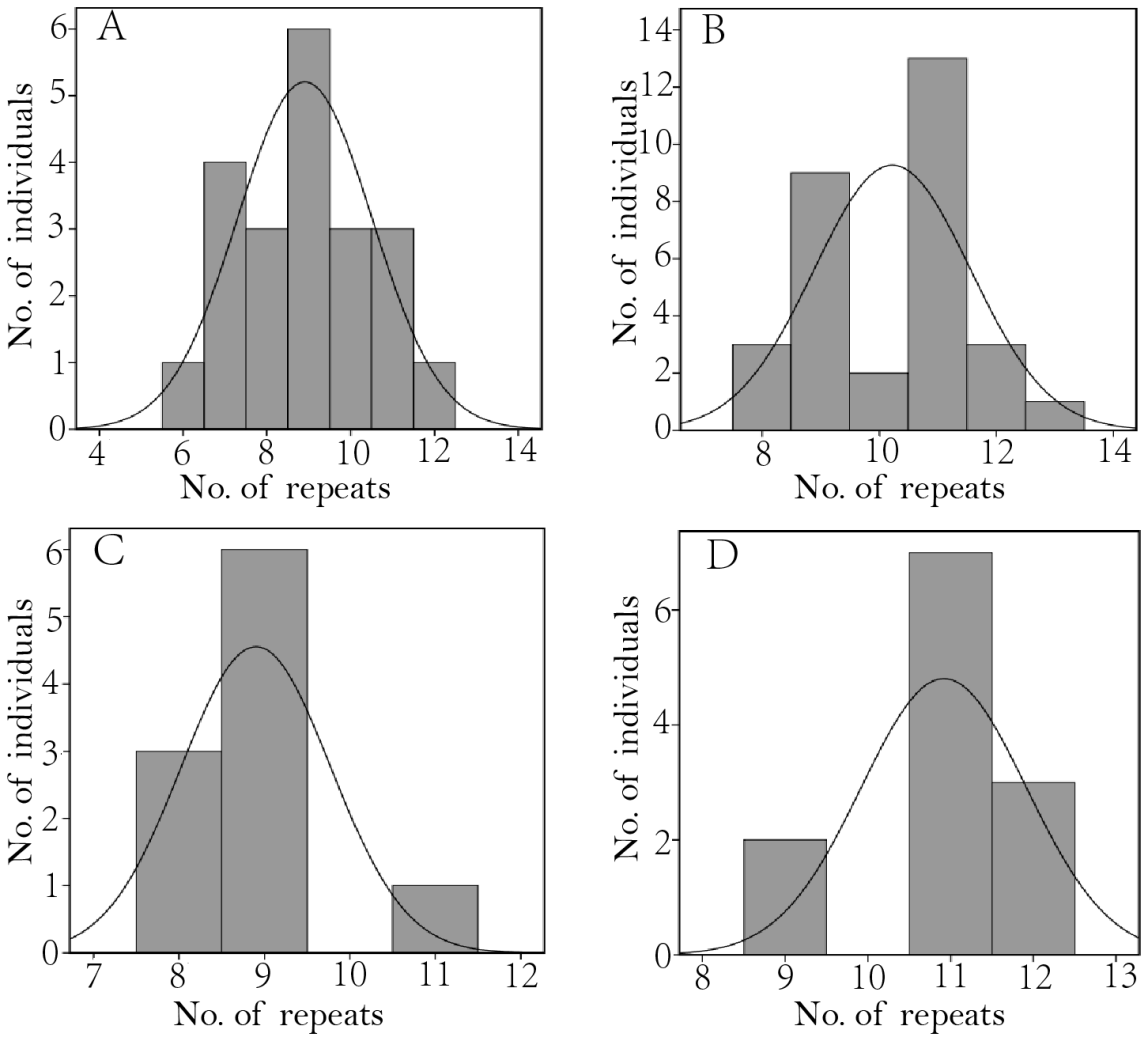
Histograms of the distribution of predominant fragments with different numbers of repeat. (A) 21 wild birds, (B) 31 captive birds, (C) Ten offspring of the mating groups 3, 5 and 6 with their mother carrying a predominant fragment of nine repeats, (D) 12 offspring of the mating group 1, 2 and 7 their mother carrying a predominant fragment of 11 repeats.

The museum foot pad specimen had a predominant fragment at 254 bp peak with 12 repeats. Among the 60 captive and wild birds, one captive bird of A-16 from No. 4 mating group carried three predominant fragments with 11, 12 and 13 repeats, one captive (A-30) and six wild birds had two predominant fragments carrying seven to 13 repeats while the rest 31 captive and 21 wild samples showed a single predominant fragment with number of repeats ranging from six to 13. The predominant fragment with nine repeats was the commonest among 21 wild birds across 19 nests while those with six, seven, eight, 10, 11 and 12 repeats were present at relatively low frequencies (Tables 1 and 2). A ‘one sample Kolmogorov-Smironv test’ implemented in the SPSS 19.0 (IBM 2010) realized that the assignments of different predominant fragments among the 21 wild samples seems to have followed a pattern in normal distribution (*P = *0.783, Figure 4A), which fitted well to the scenario of a stepwise mutation model that involves multi-step changes due to ‘replication slippage’ of a perfect microsatellite evolution (see Fig. 3 in [51] and Figure 1b in [52]).

**Table 2:** Number of repeats in predominant fragments in 27 wild Crested Ibis.

Single repeat		Two repeats	
No. ofrepeats	IDof birds	No.of repeats	ID of birds
6	C-08	7, 8	C-21
7	C-13, C-22, C-33, C-59	7, 9	C-26
8	C-01, C-34, C-52	9, 10	C-56, C-85
9	C-18, C-31, C-36, C-38, C-62, C-71	9, 12	C-37
10	C-11, C-40, C-43	9, 13	C-29
11	C-54, C-55, C-78		
12	C-23		

C-54, C-55 and C-56 are siblings.

### Transmission of the Length Polymorphism and Heteroplasmy

A first examination of the distribution pattern of frequencies of different predominant fragments among the 31 captive birds revealed a significant deviation from the normal distribution (*P = *0.024, Figure 4B). However, when the data were sorted out following the pedigree information of two major maternal lineages carrying predominant fragments with nine or 11 repeats in three mating groups each (Table 1), a match to the expected normal distribution was detected in the two lineages of either 10 progenies with their mother carrying a predominant fragment of nine repeats (*P = *0.162, Figure 4C) or 12 descendants with their mother having a predominant fragment of 11 repeats (*P = *0.079, Figure 4D).

**Figure 5:**
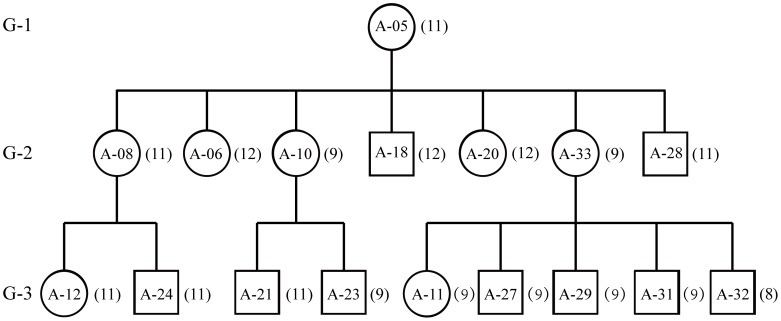
Pedigree chart represents three generations with 17 maternally-related individuals in family No. 1. G-1, G-2 and G-3 indicate the generations. Squares stand for males and circles for females. Numbers in brackets right to the squares or circles are the number of repeats in every sample.

To understand the inheritance mode of these intensive length polymorphisms and heteroplasmy in mtDNA control region, the pedigree records of 33 birds among three families were fully validated with the genotyping data of the eight microsatellite DNA markers (data not shown). Seventeen maternally-related birds in family No. 1 (Figure 5) across three generations showed that the types and/or combinations of predominant fragments, defined by different numbers of repeats, did not follow the rule of strict maternal inheritance across all mother-offspring pairs. For example, although the matriarch bird A-05 had a single predominant fragment with 11 repeats, only two out of her seven progenies shared the same predominant fragment but the remaining two and three descendants carried predominant fragments with nine and 12 repeats, respectively. Such irregular transmission was further detected in two out of the seven offspring in the third generation reproduced each by A-10 and A-33 shared the predominant fragment of nine repeats. Further assessment of the genotyping profiles showed that the secondarily predominant fragments with 11 repeats in A-10 and eight repeats in A-33 turned out to be the single dominant peaks in A-21 and A-32, respectively. In addition, four breeding males carrying different predominant genotypes from their mating females in families No. 1 and 2 allowed us to examine possible paternal leakage [53], [54], [55]. No shared predominant fragment was identified in nine out of the 11 father-offspring pairs (Table 1), thus a probable exclusion of paternal leakage in Crested Ibis mtDNA, though the evidence was not inclusive.

## Discussion

### Lack of Structural Variation in the mtDNA Control Region of Crested Ibis

As shown in Table 3, the single control region sequence obtained from this study was identical to five homologous sequences of the first part of control region (936 bp) obtained from the captive Crested Ibis population in China (accession no. AB104903–AB104907 at http://www.ncbi.nlm.nih.gov/nuccore/) but different by two nucleotides from the reference sequence (NC_008132) derived from ‘Midori’ which was one of the last survived birds in Japan. It is not clear how this complete mtDNA sequence of ‘Midori’ was generated. If the data was reliable, it may indicate the lost of a different maternal lineage once present in the Crested Ibis population in Japan.

**Table 3:** Nucleotide variation in mtDNA D-loop sequences of Crested Ibis.

Sequence/haplotype (samples)	Sequencing method	Nucleotide position					Reference
		209	240	438	532	695	
**JX853815** (27 wild and 10 captive birds)	PCR and direct sequencing	A	C	C	T	C	This study
**NC_008132** (Midori, the last wild bird in Japan)	n/a	G	T	.	.	.	http://www.ncbi.nlm.nih.gov/nuccore/NC_008132
**AB104903–AB104907** (the five birds weredescendants of Chinese captive population)	n/a	.	.	.	.	.	http://www.ncbi.nlm.nih.gov/nuccore/
**Haplotype 1** (6 wild and 20 captive birds)	PCR and cloning	–	–	–	.	.	Zhang et al. (2004) [34]
**Haplotype 2** (4 wild and 6 captive birds)	PCR and cloning	–	–	–	C	.	Zhang et al. (2004) [34]
**Haplotype I** (9 captive birds)	PCR and direct sequencing*	–	–	.	.	.	Zhang (2004) [56]
**Haplotype II** (8 captive birds)	PCR and direct sequencing*	–	–	A	.	G	Zhang (2004) [56]
**Haplotype III** (2 captive birds)	PCR and direct sequencing*	–	–	A	.	.	Zhang (2004) [56]
**Haplotype IV** (23 captive birds)	PCR and direct sequencing*	–	–	.	.	G	Zhang (2004) [56]
**One haplotype** (7 captive birds)	PCR and direct sequencing	–	–	.	.	.	Lei et al. (2004) [57]

n/a means no information on how these sequences were generated. * represents no information on how the PCR products were sequenced. A dash means that the nucleotide was not available. A dot represents the nucleotide identical to JX853815.

This new sequence was also identical to the major control region haplotype 1 but different from the minor haplotype 2. Sequence fragments from both haplotypes, which represented the 36 birds of captive and wild Crested Ibis populations in China, were cloned from a 563 bp long fragment adjacent to the repeat region [34]. A surprisingly high control region diversity with four haplotypes (298 bp) defined by two transversions was identified in 42 captive birds [56]. However, the haplotype I identical to the sequence obtained in this study was reported to be present in nine out of the 42 samples in Zhang [56] and in all seven birds in Lei et al. [57]. In fact, all the samples for these three studies were derived from the same genetic origin of the remnant ancestral population in China [4]. Thus a maximum of two different mtDNA lineages, assuming one each from the two maternal ancestors, are expected in the restored populations. The single transition reported by Zhang et al. [34] could be true for 26 birds in captivity and 10 individuals in the wild sampled from 1990 to 2000. The four haplotypes I–IV identified using the primers L438 and H1248 [56], which were claimed from Genovart et al. [58] but cited with different sequences, clearly had some errors. The same dataset was further mistakenly quoted as to have five haplotypes defined by three polymorphic nucleotides [59]. Our samples in this study had the largest number of birds from the wild (27 from 25 nests) while the 10 parental birds in captivity fully represented all maternal lineages of all 33 birds across three families. The single haplotype of mtDNA sequence covering the hyper-variable segment in the control region clearly demonstrated the absence of structural genetic diversity in mtDNA genome among the current Crested Ibis populations in China.

### Function, Origin and Inheritance of the mtDNA Length Polymorphism and Heteroplasmy in Crested Ibis

Similar to the repeat motif in (CA_3_CA_2_CA_3_)n identified among all the captive and wild Crested Ibis, such short CA-type tandem repeats located in the control region close to the tRNA-Phe region have been observed in several bird species of Alcidae, Laridae, Scolopacidae, Spheniscinae and Threskiornithidae families [19], [41], [60], [61], [62], [63], which were all thought to be related to other groups in the order Ciconiiformes following the Sibley-Ahlquist taxonomy [64]. However, the former three families are now placed in the order Charadriiformes, the forth in Sphenisciformes and the fifth in Pelecaniformes [39]. These changes seem to suggest a deep common ancestry of this type of the tandem repeats. The control region contains the replication origin for heavy strand and the promoters for transcription of both heavy and light strands [65], [66]. In human, certain mutations in the control region were accumulated with aging in specific tissues and considered to have been subject to climatic selection [67], [68]. Munwes et al. [47] studied the length heteroplasmy of a 75 bp long tandem repeat in the control region of eastern spadefoot toads (*Pelobates syriacus*) distributing from edge to core with different environmental conditions. They found that the number of repeats and level of heteroplasmy increased from edge to core, suggesting that the control region may not be neutral but under selection. Length heteroplasmy spanning the functional regions such as termination associated sequences (TAS) and light and heavy strand promoter sites (LSP and HSP, respectively) was detected in several species [14], [41], [43]. The putative TAS and conserved sequence blocks were mapped to the 5′-end to middle range of the complete control region sequence of Crested Ibis; therefore this repeat motif was believed to be an evolutionary outcome of a simple perfect microsatellite DNA structure without immediate function but the effect of possible genetic hitch-hiking on its inheritance could not be ruled out as well [69].

It is believed that the *Taq* polymerase slippage rate is inversely related to repeat motif length in microsatellites [52], [70], [71]. No stutter band was observed at chicken LEI0258 locus carrying compound repeat motifs of similar DNA sequences: ‘CTTTCCTTCTTT’ and/or ‘CTATGTCTTCTTT’ [72]. Therefore it is postulated that the stutter PCR products cannot readily explain such extensive length polymorphism and resultant heteroplasmy present in the mtDNA control region of Crested Ibis. On the other hand, recombination may have played a role to facilitate the length polymorphism among these heteroplamic mtDNA genomes [73], [74]. Multiple peaks ranging six to 13 of repeat motifs detected from the museum sample also indicated that these phenomena were not transient [73] but persistently maintained in this species for around 30 generations (assuming a three-year generation interval [6]) over the past century.

Our results showed the patterns and degrees of length polymorphism and heteroplasmy shifted in 10 out of the 23 mother-offspring pairs with the second and/or third predominant fragments of mother’s genotypes turned to be the first and/or second predominant peaks. These shifts were also observed among 17 siblings from four mating groups (Table 1). Such great shifts in transmission of different segregations across siblings and generations can be explained by a sufficiently rapid genetic drift resulted from a moderately narrow bottleneck, which considers that only small proportion(s) of the different mitochondrial genomes (e.g. carrying intensive length variations in this case) repopulate the offspring of next generation from the germ line [21], [22], [25], [26], [27], [75]. No reduction of mtDNA content in early primordial germ cells [76], [77] but the relaxed amplification of a subpopulation of mtDNA genomes in early oogenesis [23] or during postnatal folliculogenesis [78] in mice or by the end of oogenesis in fish [32] is suggested to have contributed to the random mtDNA segregation [31]. However, the differences in the properties of heteroplasmic mtDNA structures (point mutation v.s. length variation) and in the cleavage patterns of embryos between fishes (discoidal meroblastic), birds (discoidal meroblastic) and mammals (rotational holoblastic) require further investigation on how the bottleneck influences the transmission of mtDNA mutations in birds. The rapid segregation of intensive mtDNA length polymorphism and heteroplasmy firstly detected in the highly inbred Crested Ibis populations carrying no structural mtDNA diversity offers an opportunity for further investigation of the mechanism of such bottleneck responsible for random mtDNA segregation in birds.
